# Metabolic Inhibition Induces Transient Increase of L-type Ca^2+^ Current in Human and Rat Cardiac Myocytes

**DOI:** 10.3390/ijms20061501

**Published:** 2019-03-26

**Authors:** Rimantas Treinys, Giedrius Kanaporis, Rodolphe Fischmeister, Jonas Jurevičius

**Affiliations:** 1 Institute of Cardiology, Lithuanian University of Health Sciences, Kaunas LT-50162, Lithuania; rimantas.treinys@lsmuni.lt (R.T.); giedrius_kanaporis@rush.edu (G.K.); 2 INSERM UMR-S 1180, Univ Paris-Sud, Université Paris-Saclay, Châtenay-Malabry F-92296, France; rodolphe.fischmeister@inserm.fr

**Keywords:** L-type Ca^2+^ current, metabolic inhibition, FCCP, heart, cardiac myocytes, sarcoplasmic reticulum, calcium dependent inactivation

## Abstract

Metabolic inhibition is a common condition observed during ischemic heart disease and heart failure. It is usually accompanied by a reduction in L-type Ca^2+^ channel (LTCC) activity. In this study, however, we show that metabolic inhibition results in a biphasic effect on LTCC current (I_CaL_) in human and rat cardiac myocytes: an initial increase of I_CaL_ is observed in the early phase of metabolic inhibition which is followed by the more classical and strong inhibition. We studied the mechanism of the initial increase of I_CaL_ in cardiac myocytes during β-adrenergic stimulation by isoprenaline, a non-selective agonist of β-adrenergic receptors. The whole-cell patch–clamp technique was used to record the I_CaL_ in single cardiac myocytes. The initial increase of I_CaL_ was induced by a wide range of metabolic inhibitors (FCCP, 2,4-DNP, rotenone, antimycin A). In rat cardiomyocytes, the initial increase of I_CaL_ was eliminated when the cells were pre-treated with thapsigargin leading to the depletion of Ca^2+^ from the sarcoplasmic reticulum (SR). Similar results were obtained when Ca^2+^ release from the SR was blocked with ryanodine. These data suggest that the increase of I_CaL_ in the early phase of metabolic inhibition is due to a reduced calcium dependent inactivation (CDI) of LTCCs. This was further confirmed in human atrial myocytes where FCCP failed to induce the initial stimulation of I_CaL_ when Ca^2+^ was replaced by Ba^2+^, eliminating CDI of LTCCs. We conclude that the initial increase in I_CaL_ observed during the metabolic inhibition in human and rat cardiomyocytes is a consequence of an acute reduction of Ca^2+^ release from SR resulting in reduced CDI of LTCCs.

## 1. Introduction

The L-type Ca^2+^ channels (LTCCs) provide Ca^2+^ for the initiation and regulation of cell contraction and thus play a main role in cardiac muscle contraction. During normal cardiac excitation–contraction coupling, a relatively small amount of Ca^2+^ enters the cell via the L-type current (I_CaL_) and this triggers the release of a larger amount of Ca^2+^ from the sarcoplasmic reticulum (SR) via ryanodine receptors (RyRs) in a process called Ca^2+^-induced Ca^2+^ release [1]. The increase of intracellular Ca^2+^ concentration in the vicinity of closely associated Ca^2+^ channels and RyRs leads to calcium dependent inactivation (CDI) of LTCCs [2] and reduction of I_CaL_ [3]. Hence, the SR participates in regulation of the activity of plasmalemmal L-type Ca^2+^ channels [4]. The close functional interaction between plasmalemma and SR means that LTCCs and RyRs can change the activity of each other and alter the balance of cellular Ca^2+^, which directly activates the contraction of myofilaments. The mechanisms of Ca^2+^ regulation in diseased cardiac cells may be disordered. Malfunction of LTTCs and RyRs interaction may affect the contractility of myocardium and, as a consequence, the main function of the heart, to pump blood, may be altered.

Metabolic inhibition is commonly observed during ischemic heart disease and heart failure and is associated with biphasic changes in action potential (AP) duration in heart cells and whole heart [5,6]. Numerous studies show a reduction in cardiac LTCC activity and I_CaL_ during metabolic inhibition, however the mechanisms involved are not clear [7,8,9,10,11]. In ischemic heart failure, the cardiac β-adrenergic receptors are downregulated, partly because of the decreased number of the receptors and partly because of internalization and desensitization of the receptors, however also because the sympathetic system is activated and the level of catecholamines is increased [12,13]. Thus, we decided to investigate the effect of metabolic inhibition on I_CaL_ during β-adrenergic stimulation in human atrial and ventricular myocytes and rat ventricular myocytes. Surprisingly, we found that metabolic inhibition causes an acute transient increase of I_CaL_ amplitude followed by a rapid reduction. This is consistent with the most recent study demonstrating AP prolongation during metabolic inhibition in the rabbit heart [6], however is in contrast with what we reported recently in frog cardiomyocytes, where metabolic inhibition only suppresses I_CaL_ without the initial increase [11]. We explored the mechanisms responsible for the initial increase in I_CaL_ during metabolic inhibition. Our results point to a modulation of the Ca^2+^-dependent inactivation of LTCCs by the RyRs mediated Ca^2+^ release.

## 2. Results

### 2.1. Effect of Metabolic Inhibition on I_CaL_ in Human Atrial Myocytes

The first series of experiments were set to determine the effect of metabolic inhibition on I_CaL_ in human atrial myocytes. The cardiac myocytes for the first series of experiments were derived from five patients. Metabolic inhibition was induced by the application of mitochondrial uncoupler carbonyl cyanide-*p*-trifluoromethoxyphenylhydrazone (FCCP) to isoprenaline (a non-selective agonist of β-adrenergic receptors, ISO) stimulated human atrial cells and changes of I_CaL_ were monitored. Surprisingly, in most of the tested human atrial cells, FCCP induced a biphasic effect on I_CaL_, i.e., a rapid initial stimulation of I_CaL_ that was followed by a strong inhibition of I_CaL_.

**Figure 1:**
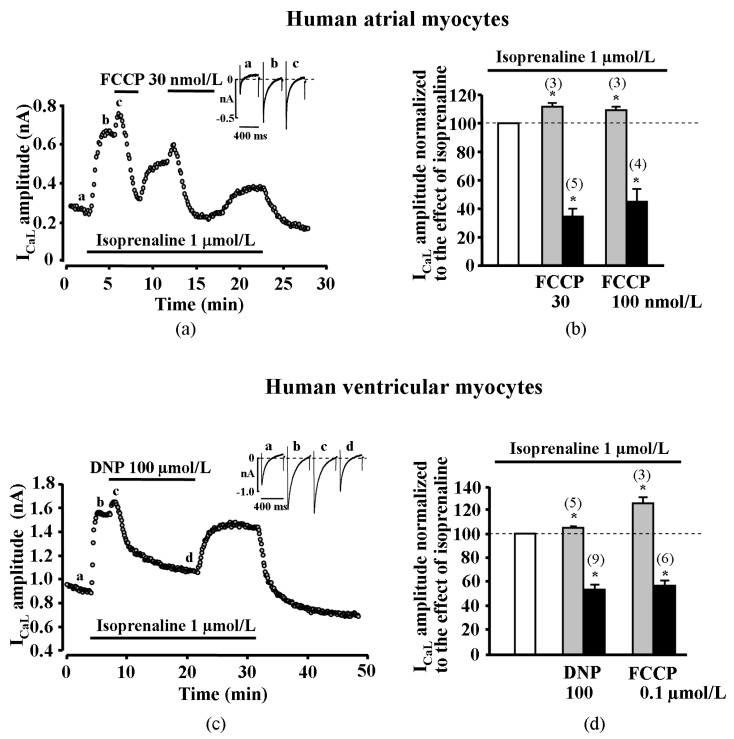

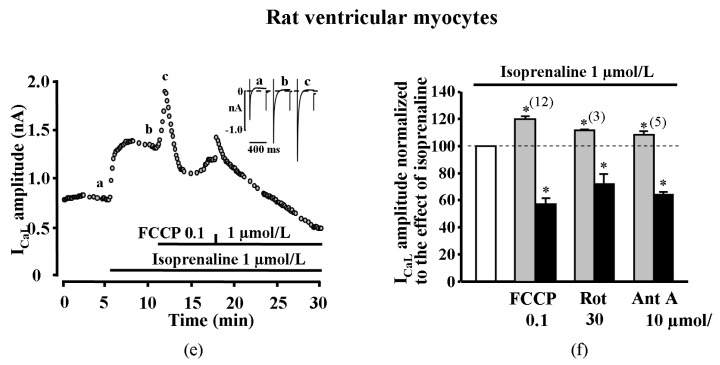
Effect of metabolic inhibition on isoprenaline stimulated I_CaL_ in human and rat cardiomyocytes. (**a**) Effect of FCCP on isoprenaline (ISO)-stimulated I_CaL_ in human atrial cell. Traces of I_CaL_ shown in the panel were recorded at the times indicated by the corresponding letters on the main graph. (**b**) Peak amplitude of I_CaL_ during exposure of ISO-stimulated human atrial cells to FCCP. (**c**) Effect of 2,4-Dinitrophenol (DNP) on ISO-stimulated I_CaL_ in human ventricular cell. Traces of I_CaL_ shown in the panel were recorded at the times indicated by the corresponding letters on the main graph. (**d**) Peak amplitude of I_CaL_ during exposure of ISO-stimulated human ventricular cells to DNP and FCCP. (**e**) Effect of FCCP on ISO-stimulated I_CaL_ in rat ventricular cells. Traces of I_CaL_ shown in the panel were recorded at the times indicated by the corresponding letters on the main graph. (**f**) Peak amplitude of ISO-stimulated I_CaL_ during exposure of rat ventricular cells to various inhibitors of oxidative phosphorylation. White bars in panels (**b**), (**d**) and (**f**) represent 100% of ISO stimulation, grey bars represent transient stimulation of I_CaL_ and black bars represent suppression of I_CaL_ during metabolic inhibition. Values are presented as means ± SEM for the number of cells indicated in parentheses. * *p* < 0.05 versus ISO alone. DNP—2,4-Dinitrophenol, Rot—rotenone, Ant A—antimycin A.

The initial stimulation of I_CaL_ induced by FCCP was recorded in six out of nine (~67%) tested human atrial myocytes. In these cells, the application of 30 or 100 nmol/L of FCCP resulted in a transient increase of ISO-stimulated I_CaL_ by 11.4 ± 2.5% (*n* = 3, *p* < 0.05, 2 patients) and 9.0 ± 2.8% (*n* = 3, *p* < 0.05, 2 patients), respectively. In all human atrial myocytes tested, metabolic inhibition led to a final suppression of I_CaL_ by 65.5 ± 5.6% (*n* = 5, *p* < 0.05, 2 patients) and 55.8 ± 9.8% (*n* = 4, *p* < 0.05, 3 patients) with 30 and 100 nmol/L FCCP, respectively (Figure 1a,b).

### 2.2. Effect of Metabolic Inhibition on I_CaL_ in Human Ventricular Myocytes

The next series of experiments were set to determine the effect of metabolic inhibition on I_CaL_ in human ventricular myocytes. The ventricular myocytes for these series of experiments were derived from eight patients. We performed experiments in human ventricle myocytes, applying metabolic inhibitors FCCP and 2,4-Dinitrophenol (DNP) on ISO (1 μmol/L) stimulated cells. We found that FCCP induced a rapid initial stimulation of I_CaL_ in three out of six ventricular myocytes (five patients). Further, 100 nmol/L of FCCP increased ISO-stimulated I_CaL_ by 24.8 ± 5.3% (*n* = 3, *p* < 0.05). The reduction of I_CaL_ induced by FCCP was registered in all tested cells, i.e., I_CaL_ was reduced by 42.5 ± 3.5% (*n* = 6, *p* < 0.05). Another uncoupler, DNP, also evoked a transient increase of ISO-stimulated I_CaL_ in five ventricular myocytes from nine (three patients). Further, 100 µmol/L of DNP increased ISO-stimulated I_CaL_ by 5.0 ± 0.9% (*n* = 5, *p* < 0.05). The reduction of I_CaL_ by 44.8 ± 4.4% induced by DNP was registered in all (*n* = 9, *p* < 0.05) human ventricular myocytes (Figure 1c,d). The initial stimulation of I_CaL_ induced by uncouplers FCCP and DNP was recorded in eight out of 15 (~53%) tested human ventricular myocytes.

### 2.3. Transient Increase of I_CaL_ is Also Elicited in Rat Cardiac Myocytes

While I_CaL_ suppression during metabolic inhibition was previously demonstrated by numerous studies [7,8,9,10], the transient increase in I_CaL_ amplitude during metabolic inhibition in cardiac myocytes has not previously been reported. To determine if this phenomenon is specific to human cardiomyocytes, we reproduced the same type of experiments in rat ventricular myocytes. Application of FCCP (100 nmol/L) in ISO-stimulated cells induced a transient increase in I_CaL_ by 20.2 ± 2.7% (*n* = 12, *p* < 0.05, four animals) that was followed by I_CaL_ reduction by 42.4 ± 4.6% (*n* = 12, *p* < 0.05) (Figure 1e,f). Higher concentrations of FCCP like 300 nmol/L (not shown) and 1 μmol/L (Figure 1e) also induced a biphasic effect in rat ventricular myocytes.

To confirm that the effects of uncouplers on I_CaL_ were due to metabolic inhibition, effects of other metabolic inhibitors were also tested. Rotenone (30 µmol/L), an inhibitor of the complex I of the mitochondrial respiratory chain, also caused an initial increase of ISO-stimulated I_CaL_ by 11.4 ± 0.2% (*n* = 3, *p* < 0.05, 2 animals) and later reduced I_CaL_ by 28.2 ± 7.6% (*n* = 3, *p* < 0.05). Antimycin A (10 µmol/L), an inhibitor of the complex III of the mitochondrial respiratory chain, initially increased ISO stimulated I_CaL_ by 8.3 ± 2.3% (*n* = 5, *p* < 0.05) and decreased by 35.5 ± 1.9% (*n* = 5, *p* < 0.05, 3 animals, Figure 1f, Supplementary Figure S1). The initial transient stimulation of I_CaL_ was recorded in all tested rat cardiomyocytes during the early phase of metabolic inhibition regardless of the metabolic inhibitor used. The effects of metabolic inhibitors in cardiac cells are summarized in Supplementary Table S1.

### 2.4. Effect of Metabolic Inhibition on I_CaL_ Kinetics

To get an insight on the possible mechanisms of I_CaL_ increase, we analyzed the effect of metabolic inhibition on time dependent inactivation of I_CaL_ in ISO stimulated cells. The time dependent inactivation of I_CaL_ in isolated adult rat ventricular myocytes was determined by bi-exponential fitting to the decay of current (see Methods) and by characterizing two kinetically distinct fast (I_CaL,1_) and slow (I_CaL,2_) current components [14]. Double exponential fit to the decay phases of I_CaL_ revealed that during the transient increase of I_CaL_ with FCCP (100 nmol/L) in ISO stimulated cells, the time constant of the fast component τ_1_ increased from 5.3 ± 0.3 ms to 7.9 ± 1.1 ms (*n* = 12, *p* < 0.05), while the time constant of the slow component τ_2_ decreased from 48.7 ± 2.9 ms to 42.6 ± 2.5 ms (*n* = 12, *p* < 0.05). The changes in the amplitude of fast and slow components of I_CaL_ were relatively small. During the inhibitory phase when the effect of FCCP reached steady-state, τ_1_ and τ_2_ were 13.8 ± 2.1 ms (*n* = 12, *p* < 0.05) and 42.7 ± 3.6 ms (*n* = 12), respectively, meanwhile the amplitude of both components of I_CaL_ were reduced. The I_CaL_ curves are presented in Supplementary Figure S2. These data show that in the presence of FCCP, there is a significant increase in τ_1_, suggesting a diminished calcium dependent inactivation (CDI) of the channels.

**Figure 2:**
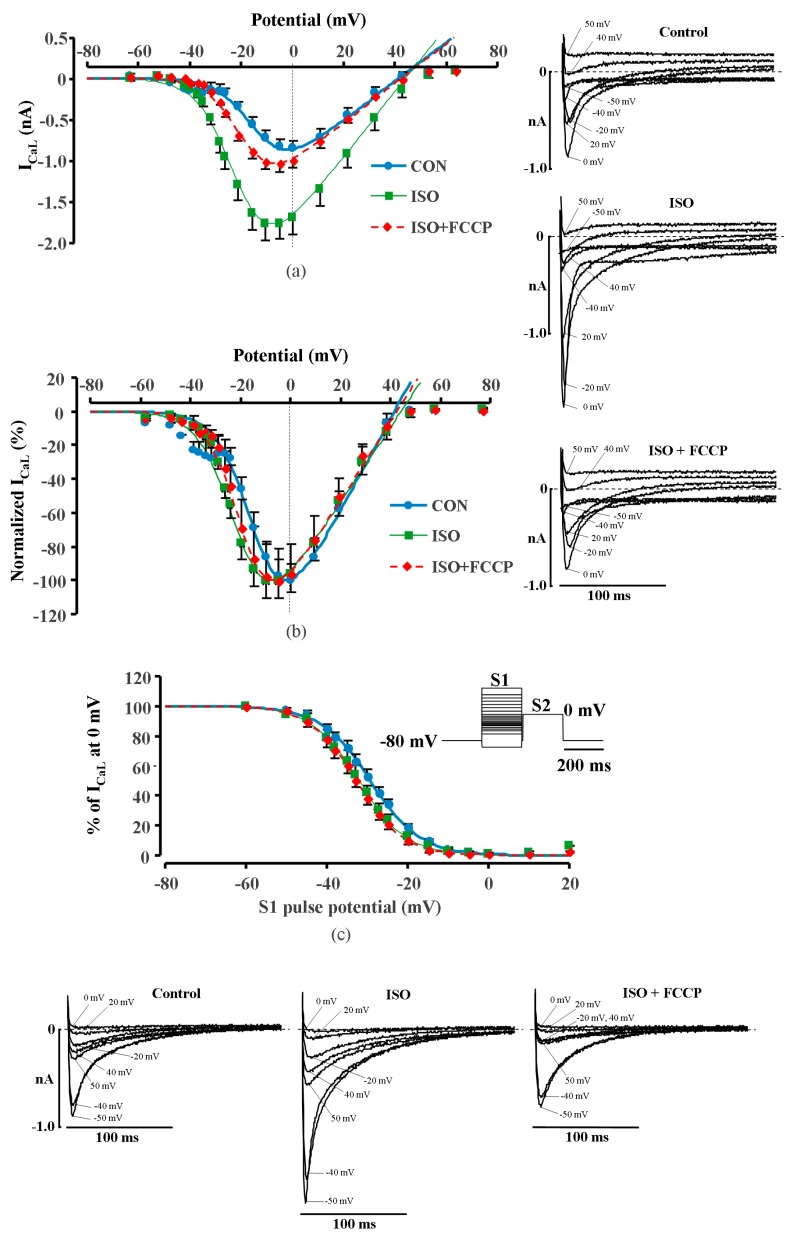
Current–voltage relationship and inactivation of I_CaL_ in rat ventricular cells. (**a**) Current–voltage (I–V) relationship, (**b**) normalized I–V relationship. Insets on the right: I–V measurements (representative of traces of I_CaL_ registered during S1 in control, in ISO stimulated cell and in the presence of FCCP). (**c**) Inactivation curves of I_CaL_ under basal conditions, during ISO (1 μmol/L) stimulation and during exposure to FCCP (100 nmol/L) in the presence of ISO in rat ventricular cells. Inset: Double-pulse protocol used for the inactivation curves (see Methods for details). I_CaL_ peak amplitude during S2 pulse is expressed as a percentage of I_CaL_ measured at 0 mV without conditioning pulse S1 and plotted as a function of S1 pulse potential. Points represent experimentally measured values; lines represent values calculated with Boltzmann equation. Insets below: Inactivation of I_CaL_ (representative traces of I_CaL_ registered during S2 in control, in ISO stimulated cell and in the presence of FCCP).

We also measured the current–voltage relationship (I–V) and the steady–state inactivation of I_CaL_ in rat ventricular cells (Figure 2). Averaged data of I_CaL_ I–V curves (Figure 2a and normalized data in Figure 2b) and inactivation (Figure 2c) in control conditions, in the presence of ISO and in the presence of ISO + FCCP, are shown. The points in the figure represent experimental recorded data and the I–V curves were fitted to the Boltzmann equation (see Methods). Derived values for V_1/2_ in control, ISO and FCCP were −13.26 ± 0.87 mV, −20.71 ± 0.81 mV and −18.80 ± 0.99 mV and k was 6.9 ± 0.4, 7.1 ± 0.6 and 6.5 ± 0.2 (*n* = 3), respectively. As expected, ISO significantly shifted I–V curve to the left [15] by > 7 mV (*p* < 0.05) while FCCP had no significant effect on the I_CaL_ I–V relationship. The fit of the steady–state inactivation curves for control, ISO and FCCP led to V_1/2_ values of −29.20 ± 1.15 mV, −32.05 ± 1.60 mV and −32.75 ± 1.42 mV, respectively, and k was 6.2 ± 0.1, 5.9 ± 0.3 and 5.7 ± 0.1 (*n* = 3), respectively. Again, ISO significantly shifted the inactivation of I_CaL_ to the left by ≈3 mV (*p* < 0.05), however the effect of FCCP was negligible. The changes of curve steepness k under the influence of ISO or FCCP were insignificant both for I–V and for steady–state inactivation curves. 

### 2.5. Suppression of Cytosolic Ca^2+^ Release Abolish Transient Increase of I_CaL_

The above results showed that in human and rat cardiac cells, metabolic inhibition induced initial transient increase of I_CaL_. In our previous study, we found no such stimulation during metabolic inhibition in frog cardiomyocytes [11] where SR is scarce and lacks ryanodine channels [16] and, in contrast to mammalian cells, LTCCs do not have such a strong functional relationship with RyRs. Consequently, we hypothesized that the observed increase of I_CaL_ in mammalian cells may involve metabolic sensitivity of SR and RyRs in regulation of calcium dependent inactivation (CDI) of LTCCs. To test this hypothesis, additional experiments were performed in rat cardiomyocytes in which Ca^2+^ release from SR was suppressed prior to metabolic inhibition.

**Figure 3:**
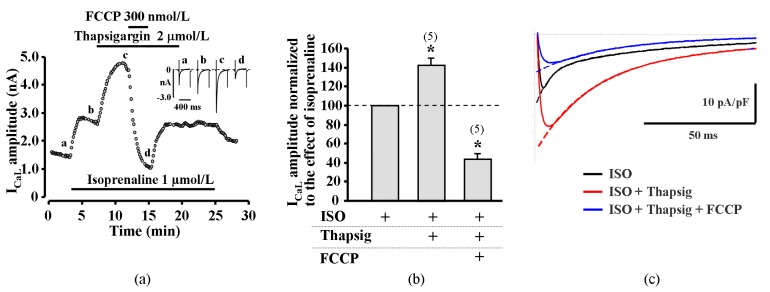
Effect of FCCP on isoprenaline stimulated I_CaL_ in rat cardiomyocytes after the suppression of cytosolic Ca^2+^ release. (**a**) A typical experiment representing the effect of FCCP on I_CaL_ in ISO-stimulated cells during exposure to thapsigargin. Traces of I_CaL_ shown in the panel were recorded at the times indicated by the corresponding letters on the main graph. (**b**) Peak amplitude of I_CaL_ during exposure of ISO-stimulated rat ventricular cells to FCCP in the presence of thapsigargin. Values are presented as means ± SEM for the number of cells indicated in parentheses. * *p* < 0.05 versus ISO alone. (**c**) The time dependent inactivation of LTCCs currents. Traces of I_CaL_ in isoprenaline (ISO, 1 µmol/L) stimulated (black line) rat cell during application of thapsigargin (Thapsig, 2 µmol/L) (red line) and metabolic inhibition by FCCP (0.1 µmol/L) (blue line). Dashed lines represent double exponential fits of I_CaL_s decay.

First, metabolic inhibition was induced after the pretreatment of myocytes with thapsigargin (2 μmol/L). Thapsigargin inhibits SR Ca^2+^ ATPase (SERCA) and impairs Ca^2+^ uptake by the SR and thus leads to the depletion of Ca^2+^ in the SR [17]. As expected, thapsigargin diminished CDI of LTCCs which led to the increase in ISO-stimulated I_CaL_ in rat ventricular myocytes to 142.1 ± 8.3%, i.e., from 17.7 ± 0.5 pA/pF to 25.1 ± 0.9 pA/pF (*n* = 5, *p* < 0.05). In the presence of thapsigargin, the initial I_CaL_ increase was completely eliminated and only an inhibitory effect of FCCP on ISO stimulated I_CaL_ was observed (*n* = 5, *p* < 0.05) (Figure 3a,b). Double exponential fit of the decay of I_CaL_ revealed that application of thapsigargin on ISO stimulated rat cardiac cells resulted in an increase of all I_CaL_ parameters. The time constants τ_1_ and τ_2_ increased from 5.5 ± 0.5 ms to 26.2 ± 2.6 ms and from 70.3 ± 4.2 ms to 88.2 ± 3.0 ms (*n* = 4, *p* < 0.05), respectively. In the presence of thapsigargin, the effect of FCCP on τ_1_ was abolished. The time constant τ_1_ in such conditions remained similar to thapsigargin treated cells and was 26.4 ± 0.5 ms, while τ_2_ was similar to ISO stimulated I_CaL_ and was 69.1 ± 3.7 ms (*n* = 4). The representative I_CaL_ traces and their fits are shown in Figure 3c. Additionally, we used an alternative approach to eliminate the CDI of LTCCs prior the metabolic inhibition by blocking RyR channels with ryanodine (10 μmol/L) in rat ventricular cells. Similar to experiments with thapsigargin, ryanodine increased ISO stimulated I_CaL_ and the application of FCCP failed to induce the initial I_CaL_ increase and, thus, only an inhibition of ISO stimulated I_CaL_ was observed (Supplementary Figure S3).

**Figure 4:**
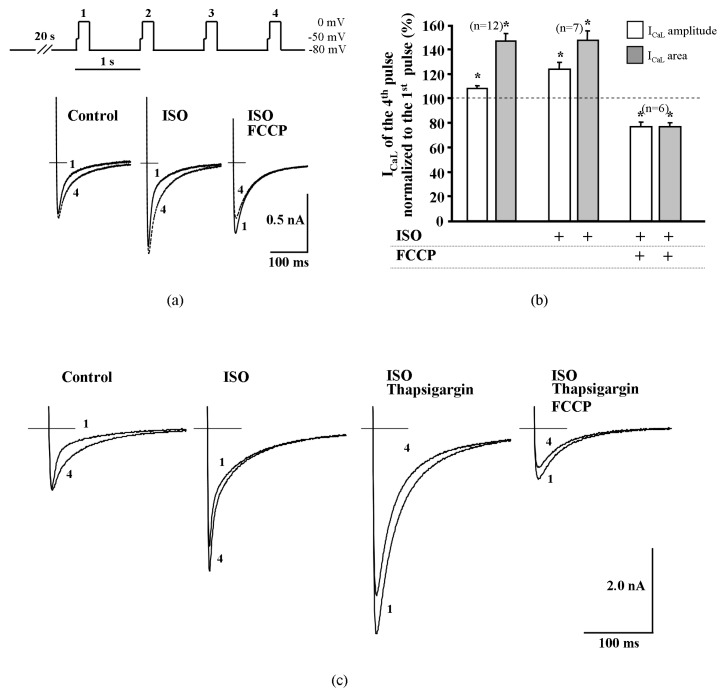
Fast facilitation of I_CaL_ in rat ventricular cells is abolished by metabolic inhibition and by the suppression of cytosolic Ca^2+^ release. (**a**) Depolarization protocol for the induction of fast facilitation of I_CaL_ shown on top. In ISO-stimulated rat ventricular cells and in control conditions, increased frequency (to 1 Hz) depolarization induced an increase of I_CaL_ amplitude and delayed the inactivation; FCCP abolished this effect in ISO-stimulated cells. (**b**) Peak amplitude and area (time integral) of I_CaL_ during the fourth depolarizing pulse in control conditions, in ISO stimulated cells and during exposure of 0.1 μmol/L of FCCP. Values are presented as means ± SEM for the number of cells indicated in parentheses. * *p* < 0.05 versus I_CaL_ of the first pulse. (**c**) Suppression of cytosolic Ca^2+^ release by thapsigargin abolished the increase of I_CaL_ amplitude itself during increased frequency depolarization and FCCP additionally reduced I_CaL_ amplitude.

The results obtained with thapsigargin and ryanodine in rat ventricular cells point to a possible role of CDI in the I_CaL_ increase during the early phase of metabolic inhibition. To further confirm this hypothesis, we measured the changes of I_CaL_ facilitation in rat cardiac cells in the absence and presence of FCCP. Increased frequency (to 1 Hz) of depolarization after a 20 s pause was used in these series of experiments. The peak amplitude of I_CaL_ and the I_CaL_ area were compared at the time of the first and fourth depolarizing pulse. In control conditions, increased stimulation frequency enhanced I_CaL_ amplitude by 8.3 ± 2.1% and the area by 46.9 ± 6.3% (*n* = 12, *p* < 0.05) during the fourth depolarizing pulse. In ISO stimulated rat cardiac cells, increasing stimulation frequency to 1 Hz also induced I_CaL_ facilitation and increased I_CaL_ peak amplitude and area by 22.2 ± 5.3% and 42.0 ± 8.6% (*n* = 7, *p* < 0.05), respectively (Figure 4a,b). Double exponential fits of I_CaL_ decay during the first and fourth depolarizing pulses revealed that the time constant of the fast component τ_1_ increased from 5.8 ± 0.5 ms to 7.3 ± 0.7 ms (*n* = 8, *p* < 0.01), while the time constant of the slow component τ_2_ decreased from 62.1 ± 4.8 ms to 51.3 ± 2.6 ms (*n* = 8, *p* < 0.01). The I_CaL_ curves are presented in Supplementary Figure S4. After treatment of rat ventricular myocytes with FCCP, I_CaL_ facilitation was no longer observed and I_CaL_ amplitude and area were reduced by 23.6 ± 4.1% and 23.1 ± 3.3% (*n* = 6, *p* < 0.05, Figure 4a,b) during the fourth depolarizing pulse, respectively.

These data indicate that pharmacological or frequency-dependent depletion of Ca^2+^ from the cytosolic Ca^2+^ store results in an increased τ_1_ and thus reduced CDI, followed by an increase of Ca^2+^ influx through LTCCs, a process that is possibly observed during the early phase of metabolic inhibition.

Depletion of Ca^2+^ from the SR by thapsigargin eliminated I_CaL_ facilitation (Figure 4c) and, during the application of I_CaL_ facilitation protocol, the amplitude and area of I_CaL_ measured during the fourth depolarizing pulse were reduced by 20.0 ± 0.6% and 22.8 ± 2.4%, respectively (*n* = 4, *p* < 0.05) when compared with the first depolarizing pulse. After the application of FCCP in ISO-stimulated and thapsigargin treated rat cells, the I_CaL_ frequency-dependent facilitation remained abolished and the amplitude and area of I_CaL_ were reduced during the fourth depolarizing pulse by 22.7 ± 1.8% and 20.0 ± 1.4%, respectively (*n* = 4, *p* < 0.05). The observation that the application of FCCP abolishes the facilitation of I_CaL_ in a similar manner as the depletion of Ca^2+^ from the SR, indicates that FCCP affects CDI of LTCCs.

### 2.6. Effect of Metabolic Inhibition on Ba^2+^ Current (I_Ba_) through L-Type Calcium Channels

**Figure 5:**
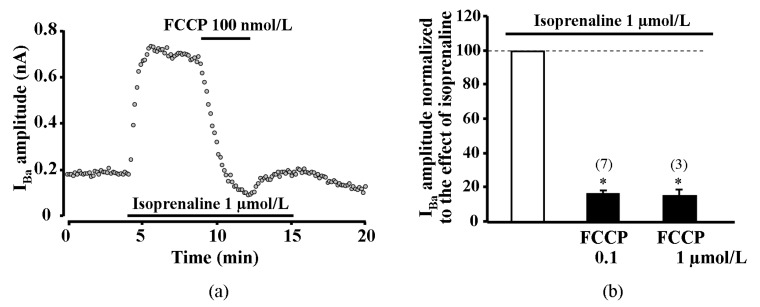
Effect of FCCP on isoprenaline stimulated Ba^2+^ current through L-type calcium channels in human atrial myocytes. Only suppression of Ca^2+^ channel current was registered during metabolic inhibition after equimolar substitution of extracellular Ca^2+^ for Ba^2+^. (**a**) A typical experiment representing the effect of FCCP on I_Ba_ in ISO-stimulated cells. (**b**) Peak amplitude of I_Ba_ during exposure of ISO-stimulated human atrial cells to FCCP. Values are presented as means ± SEM for the number of cells indicated in parentheses. * *p* < 0.05 versus ISO alone.

As an alternative approach to test if reduced CDI plays a key role in the transient potentiation of I_CaL_ during metabolic inhibition, we performed experiments in human atrial myocytes in which CaCl_2_ in the external solution was replaced by the equimolar BaCl_2_. LTCCs are permeable to Ba^2+^, however Ba^2+^ ions do not induce Ca^2+^ release from the SR and, thus, abolish CDI of LTCCs. As shown in Figure 5, metabolic inhibition induced only the suppression of Ba^2+^ current (I_Ba_) without the transient increase observed in I_CaL_. At 100 nmol/L and 1 μmol/L concentrations, FCCP suppressed the ISO-stimulated I_Ba_ by 83.3 ± 1.6% (*n* = 7, *p* < 0.05, 3 patients) and 84.5 ± 3.5% (*n* = 3, *p* < 0.05, 2 patients), respectively (Figure 5b).

## 3. Discussion

In the present study, we demonstrate that metabolic inhibition has a biphasic effect on I_CaL_ in mammalian cardiac myocytes. In most human atrial and ventricular myocytes and in all the tested rat ventricular myocytes that were stimulated by isoprenaline metabolic inhibition, this induced an initial transient increase of I_CaL_ which was followed by a strong inhibition of I_CaL_. The transient increase in ISO stimulated I_CaL_ was induced by various metabolic inhibitors (FCCP, DNP, rotenone, antimycin A), demonstrating that the observed effect is not dependent on the method used to induce metabolic inhibition. In the control experiments without metabolic inhibition, we observed only run-down of the I_CaL_ and no spontaneous increase in I_CaL_ was detected (Supplementary Figure S5). We propose that the transient I_CaL_ increase during the initial phase of metabolic inhibition is due to impaired intracellular calcium cycling and suggest that this increase in I_CaL_ is mediated by weak CDI due to metabolic failure of Ca^2+^ release from SR via RyRs. Presumably, during metabolic inhibition, the stimulation of I_CaL_ and suppression of I_CaL_ happen at the same or almost at the same time and therefore it is difficult to discriminate between these two processes. The suppression becomes dominant during exposure to the metabolic inhibitors and therefore makes evaluation of dose-response difficult and not reliable. In this study, we seek to reveal the mechanism of the transient increase of I_Ca,L_ during metabolic inhibition and did not conduct concentration–dependence experiments.

To our knowledge, I_CaL_ stimulation by metabolic inhibitors has not been previously reported in cardiac cells. McHugh and Beech (1996) [18] found a similar effect in single smooth muscle cells isolated from the basilar artery of the guinea pig where the mitochondrial uncoupler 2,4-dinitrophenol induced an initial stimulation of I_CaL_ in some of the cells, which was followed by a pronounced current inhibition. The authors have attributed this effect to a leftward shift of the Ca^2+^ channel activation curve. In our experiments, the leftward shift in the I_CaL_ I–V relationship was induced only by application of ISO which is consistent with β-adrenergic stimulation [15]. However, there was no additional shift in the I_CaL_ I–V curve during the application of FCCP (Figure 2). Therefore, the transient increase in I_CaL_ during metabolic inhibition in cardiac myocytes can not be attributed to the changes in LTCCs voltage-dependent activation.

Our recent study [11] has revealed that metabolic inhibition only exerts a strong inhibitory effect on I_CaL_ in amphibian heart cells without the initial transient increase observed here. In frog cardiomyocytes, SR is scarce and lacks ryanodine channels [16]. Thus, LTCCs are the primary source of Ca^2+^ for contraction in amphibian cardiomyocytes and Ca^2+^ release from the SR plays only a minor role, while in mammalian hearts, the SR serves as the main Ca^2+^ store and plays an important role in the regulation of the activity of LTCCs [4,19]. Therefore, we have hypothesized that impairment of Ca^2+^ induced Ca^2+^ release (CICR) leading to diminished Ca^2+^ dependent inactivation (CDI) of LTCCs might underlie the transient increase in I_CaL_ amplitude. It has previously been demonstrated that metabolic inhibition affects properties of intracellular Ca^2+^ release and decreases frequency of spontaneous Ca^2+^ waves [10,20]. The reports on the changes in SR Ca^2+^ load during metabolic inhibition are rather inconsistent as some studies have reported no change or a decrease in SR Ca^2+^ content [8,21], while others demonstrated an increase in SR Ca^2+^ load [20]. Such discrepancies in the results can be at least partially explained by the reported biphasic effect of metabolic inhibition on intracellular Ca^2+^ signaling, consisting of an initial inhibition followed by stimulation of SR Ca^2+^ release [10]. The effect of metabolic inhibition on SR may arise due to the changes in high-energy phosphate compounds and/or the increase in intracellular acidosis. Recently, we have shown that acidosis may play a key role in the suppression of LTCCs activity during metabolic inhibition [11]. The intracellular acidification may also inhibit the release of Ca^2+^ from SR as well as the reuptake of Ca^2+^ to the SR by SR Ca^2+^ ATP-ase, the activity of which may also be inhibited by deficiency of energy compounds. During metabolic inhibition, both Ca^2+^ release (RyRs) and reuptake (Ca^2+^ ATP-ase) mechanisms are inhibited to a different extent [20]. Recently, it was demonstrated that during metabolic inhibition, calcium transients (CaTs) are decreased and AP is prolonged [6]. This reduction of CaT can result in the slowdown of LTCCs inactivation and may be involved in the prolongation of AP in rabbit hearts [6]. The transient increase of I_CaL_ during ischemia may lead to early afterdepolarisation and long QT which can be followed by arrhythmias. In our study, double exponential fit to I_CaL_ curves showed a significant increase of the fast component τ_1_ during the transient increase of I_CaL_ in the presence of FCCP. It is known that the fast component of I_CaL_ inactivation is dependent on the magnitude of Ca^2+^ release from SR [2]. Thus, this also points to the possible connection between the transient increase of I_CaL_ and intracellular Ca^2+^ release during metabolic inhibition. In order to test the hypothesis that the changes of Ca^2+^ release from SR result in the potentiation of I_CaL_, we have performed a series of experiments where, prior to the application of metabolic inhibitors, SR Ca^2+^ release was suppressed. Suppression of SR Ca^2+^ release in cardiac cells reduces CICR and therefore leads to decreased CDI of LTCCs. Consequently, a significant augmentation of I_CaL_ was observed in the presence of thapsigargin or ryanodine (Figure 3 and Supplementary Figure S3). This is consistent with observations that the elimination of the CICR-dependent CDI using thapsigargin or ryanodine, significantly prolongs AP duration in rat ventricular myocytes [22,23]. When thapsigargin and ryanodine treated rat ventricular myocytes were exposed to FCCP, the initial increase of I_CaL_ was completely abolished and only pronounced suppression of I_CaL_ was observed. In addition, in the presence of thapsigargin, the effect of FCCP on the I_CaL_ inactivation fast time constant τ_1_ (a parameter that is greatly affected by CDI) was eliminated. We suggest that under these conditions, CDI of I_CaL_ is abolished and, therefore, an increase in I_CaL_ is not observed. For the same reason, there is a lack of I_CaL_ facilitation in thapsigargin treated rat cardiac cells where the decay characteristics of fast and slow components of I_CaL_ were significantly augmented and subsequent metabolic inhibition resulted only in a rapid decrease of I_CaL_ without any initial I_CaL_ increase. The finding that CDI plays a crucial role in the initial potentiation of I_CaL_ during metabolic inhibition was also supported by the experiments where CDI was eliminated by equimolar substitution of extracellular Ca^2+^ for Ba^2+^ (Figure 5). There are also evidences that mitochondria can control the local Ca^2+^ level in the micro-domain near SR ryanodine receptors and play an important role in the regulation of intracellular Ca^2+^ waves and arrhythmogenesis.

The double exponential fits and analysis of ISO stimulated I_CaL_ decay traces showed that transient I_CaL_ increase during metabolic inhibition mimics what is observed during rapid pacing induced facilitation of I_CaL_, which is well known to be related to CDI [4]. Increased frequency of stimulation resulted in a significant augmentation of the time constant of the fast component τ_1_, while τ_2_ was significantly diminished. Exactly the same changes in I_CaL_ kinetics were revealed at the stimulation phase of I_CaL_ during metabolic inhibition. These results are consistent with Tiaho et al. (1994), where they describe the decay phases of I_CaL_ at the stimulation frequency of 1 Hz.

The increase in I_CaL_ amplitude was registered not in all human cardiac myocytes and this may point to possible impairment in cells derived from pathological cardiac specimens. However, we were not able to find a relation between the transient increase in I_CaL_ (or its absence) and any pathology of the human heart. Moreover, we found that even myocytes derived from the same patient showed a different response to metabolic inhibition, i.e., in some cardiomyocytes, the transient increase of I_CaL_ was registered, while in others it was not. We suggest that the observation that transient I_CaL_ increase is induced just in some of human atrial and ventricular myocytes while all rat ventricular myocytes exhibit an increase in I_CaL_ during metabolic inhibition, can be attributed to the pathophysiological state of the particular cell as well as to differences between species. The pathophysiological state of the cell and thus the different response to metabolic inhibition may be related with attenuation or downregulation of β-adrenergic signaling in human heart cells [12,13]. Rat ventricular myocytes were isolated from the healthy animals, while human cardiomyocytes were obtained from patients of different ages and various cardiac pathologies (Supplementary Table S2). Disorganization and degradation of T-tubule network during pathophysiological remodeling of the heart is known to disrupt LTCC and RyR coupling [24] and to contribute to the variability of response in myocytes isolated from the patients. In addition, it was demonstrated that even in the healthy hearts, there are substantial differences between small rodents and large mammals in T-tubule organization and RyR distribution, with the T-tubule network being much denser and Ca^2+^ release from SR more homogeneous in rodents’ ventricular myocytes [25]. There is also evidence that mitochondria are involved in the control of the local Ca^2+^ level in the micro-domain near SR RyRs and that they play an important role in the regulation of intracellular Ca^2+^ waves and arrhythmogenesis [26]. The initial increase of I_CaL_ shows the abnormalities of Ca^2+^ cycling in heart cells during ischemia (metabolic inhibition) which may lead to the arrhythmogenic processes of the heart.

## 4. Materials and Methods

### 4.1. Isolation of Cardiomyocytes

#### 4.1.1. Human Atrial and Ventricular Myocytes

This study was carried out in accordance with the recommendations of the principles outlined in the Declaration of Helsinki, Kaunas Regional Bioethics Committee (Lithuania) with written informed consent from all subjects. All subjects gave written informed consent in accordance with the Declaration of Helsinki. The protocol was approved by the Kaunas Regional Bioethics Committee (16 November 2015 No. BE-2-40). Specimens of human heart were obtained from 16 patients undergoing heart surgery for coronary artery diseases at the Hospital of Lithuanian University of Health Sciences (Kaunas, Lithuania). Most of the patients had received a pharmacological treatment (including Ca^2+^-channel blockers, digitalis, β-AR antagonists, diuretics, ACE inhibitors, NO-donors and/or antiarrhythmic drugs) that was stopped 24 h before surgery. In addition, before and during surgery, all patients received sedatives, anesthesia and antibiotics. Information on patient group and specimens is provided in Supplementary Table S2. Dissociation of the cells was performed as described previously [27]. Briefly, the specimens of the atrial or ventricular tissue were washed in an oxygenated Ca^2+^ free Tyrode solution (in mmol/L): 136 NaCl, 5.4 KCl, 1.1 MgCl_2_, 10 HEPES, 20 taurine, 5 sodium pyruvate, 10 d-glucose, 0.3 NaH_2_PO_4_, pH 7.3 adjusted with NaOH at room temperature. Later, the tissue was cut into pieces of ~1 mm^3^ in Tyrode solution with 30 mmol/L 2,3-butanedionemonoxime added to the solution. Subsequently, a 30–40 min incubation with collagenase (type V, 200 U/mL) and protease (type XXIV, 5 U/mL) in 10 mL Ca^2+^ free BSA (5 mg/mL) containing Tyrode solution was performed at 37 °C. The solution was then replaced by 10 mL of fresh solution containing collagenase (400 U/mL) and digestion was repeated for 20–30 min. Every five minutes, a small sample of solution was examined under a microscope to evaluate the stage of cell dissociation.

#### 4.1.2. Rat Ventricular Myocytes

This study was carried out in accordance with the European Community guiding principles and recommendations of the Guide for the Care and Use of Laboratory Animals of the National Institutes of Health and UK regulations on animal experimentation [28]. The protocol was approved by the State Food and Veterinary Service of the Republic of Lithuania (24 September 2015 No. G2-34). Adult rat ventricular myocytes were obtained by retrograde perfusion from hearts of male Wistar rats (160–180 g) as described previously [29]. Briefly, rats were anesthetized with intraperitoneal injection of Pentothal and hearts were excised quickly. The hearts were retrogradely perfused at 37 °C with an oxygenated Ca^2+^ free Ringer solution (in mmol/L): 117 NaCl, 5.8 KCl, 4.4 NaHCO_3_, 1.5 KH_2_PO_4_, 1.7 MgCl_2_, 11.7 d-glucose, 10 sodium phosphocreatine, 20 taurine, 21 HEPES, pH 7.1 adjusted with NaOH at room temperature. For heart myocyte, dissociation collagenase type A (1 mg/mL, Boehringer Mannheim, Germany) was added to the Ca^2+^ free Ringer solution and the heart was perfused for 1 h. The ventricles were then separated from atria, cut to small pieces and agitated gently to dissociate the single cells.

#### 4.1.3. Electrophysiology

For electrophysiological experiments, a few drops of cells suspension were placed in a perfusion chamber mounted on an inverted microscope stage. After cells had settled to the bottom, the chamber was superfused with K^+^ free control external solution containing (in mmol/L): 127 NaCl; 10 HEPES; 20 CsCl; 4 NaHCO3; 0.8 NaH2PO4; 1.8 MgCl2; 1.8 CaCl2; 5 d-glucose; 5 sodium pyruvate; pH 7.4 adjusted with NaOH. Patch pipettes were made from glass capillaries (Drummond, Broomall, PA, USA) and had resistances of 0.9–1.2 MΩ when filled with control internal solution. The myocytes were dialysed with control internal solution composed of (in mmol/L): 140 CsCl, 5 EGTA, 4 MgCl_2_, 0.062 CaCl_2_, 5 creatine phosphate disodium salt, 3.1 Na_2_ATP, 0.42 Na_2_GTP and 10 HEPES; pH to 7.3 was adjusted with CsOH. The whole-cell patch–clamp technique was used to record the I_CaL_ in human and rat cardiac myocytes as described previously [30]. Ionic currents through LTCCs were registered every 8 s. I_CaL_ facilitation was generated after 20 s of rest applying four depolarizing pulses from −80 mV holding potential to 0 mV for 200 ms (frequency 1 Hz). Tetrodotoxin (30 μmol/L, Latoxan, Rosans, France) and a 50 ms prepulse to −50 mV before every depolarizing pulse were used to eliminate sodium currents. Inward peak currents were measured as the difference between the maximal inward current amplitude and the current at the end of the test pulse [27].

Equilibrium for steady-state frequency-dependent facilitation of I_CaL_ is reached after four stimulations [31], therefore I_CaL_ facilitation was assessed comparing the first I_CaL_ trace with the fourth trace at 1 Hz. The peak of I_CaL_ and the I_CaL_ area, an integral of I_CaL_ for 200 ms (pA x ms), were registered at the time of the first and fourth depolarizing pulse. The time integral of I_CaL_ was registered in part of the experiments to elucidate the origin of I_CaL_ increase during metabolic inhibition and to compare the initial effect of FCCP with frequency-dependent facilitation and reduced CDI.

To determine the current–voltage (I–V) relationship and inactivation of I_CaL_, a double pulse voltage-clamp protocol was applied every 4 s (see insert in Figure 2c). During the first pulse S1, the membrane potential was set at membrane potentials ranging from −100 to +100 mV for 200 ms. S1 pulse was followed by a 3 ms repolarization to the −80 mV holding potential and then a depolarizing pulse S2 to 0 mV was applied for 200 ms.

All experiments were performed at room temperature (18–24 °C) and the temperature did not change by more than 1°C during an experiment.

#### 4.1.4. Chemicals and Stock Solutions

All drugs and chemicals were from Sigma-Aldrich (Schnelldorf, Germany) if not specified otherwise. All drugs tested in patch-clamp experiments were solubilized in experimental solutions just before application onto the cell studied, i.e., only fresh solutions were tested. To block oxidative phosphorylation stock solutions of carbonyl cyanide-*p*-trifluoromethoxyphenylhydrazone (FCCP, 100 μmol/L), 2,4-Dinitrophenol (DNP, 100 mmol/L) in ethanol, rotenone (100 mmol/L) and antimycin A (100 mmol/L) in DMSO were used. The stock solution of the SR Ca^2+^ ATP-ase inhibitor thapsigargin (10 mmol/L) was prepared in ethanol. A stock solution of ryanodine (10 mmol/L) for blocking of RyR channels was prepared in distilled water. An aqueous stock solution of the isoprenaline (ISO, 1 mmol/L) was freshly prepared every day prior to the experiments. When the stock solutions in DMSO or ethanol were used, the corresponding amount of solvent was also added to the external control solution.

#### 4.1.5. Data Analysis

Inactivation of I_CaL_ traces were best fitted by the sum of two sequential exponentials using the expression:

I(t) = I_CaL,1_ × exp(−t/τ_1_) + I_CaL,2_ × exp(−t/τ_2_), (1)

where I is the current at time t; I_CaL,1_ and I_CaL,2_ are the amplitudes and τ_1_ and τ_2_ are the time constants of the fast and slow components of I_CaL_, respectively. The fitting of I_CaL_ inactivation traces was performed using the modified protocol described by [32] in rat ventricular myocytes.

The fitting time was set from ~1 ms after the peak of inward calcium current until the end of 400 ms pulse. The steady-state current was calculated at the end of 400 ms pulse and this helped to avoid residual leak current influence to the inactivation of I_CaL_. The quality of the fits were evaluated by correlation coefficients (r) and in all of the experiments, the correlation coefficient was ≥0.985.

Steady-state inactivation curves (B_1_) and I–V traces (B_2_) were fitted with Boltzmann equations:

B_1_(V) = 1/(1 + exp((V_1/2_ − V)/k); (2)

B_2_(V) = g(V − V_rev_)/(1 + exp((V_1/2_ − V)/k), (3)

where V_1/2_ is 0.5 of the maximal value of steady-state activation and inactivation, V_rev_ is the reversal potential of Ca^2+^, g is the conductance of LTCC and k is the steepness parameter.

Data is expressed as the means ± standard errors of the mean (SEM). The significance of differences was evaluated using one-way analysis of variance (ANOVA). The significance level was set at *p* < 0.05. In the text, the “basal I_CaL_” refers to the Ca^2+^ current which was not stimulated by β-adrenergic agonist.

## 5. Conclusions

In conclusion, our data demonstrate that metabolic inhibition in mammalian cardiomyocytes causes an initial stimulation of I_CaL_ which is due to a reduction in SR function and an alleviation of the Ca^2+^-dependent inactivation of the L-type Ca^2+^ channels. These mechanisms may contribute to the formation of arrhythmias in the ischemic heart and may be targets for new treatment methods of heart pathologies in the future.

## Supplementary Materials

Supplementary materials can be found at https://www.mdpi.com/1422-0067/20/6/1501/s1.

## Abbreviations

LTCC	L-type Ca^2+^ channel
SR	Sarcoplasmic reticulum
CDI	Calcium dependent inactivation
RyRs	Ryanodine receptors
ISO	Isoprenaline
FCCP	Carbonyl cyanide-*p*-trifluoromethoxyphenylhydrazone
