# Supplementary material for: Metabolic Inhibition Induces Transient Increase of L-type Ca2+ Current in Human and Rat Cardiac Myocytes
Source: Int J Mol Sci. 2019 Mar 26;20(6):1501. doi: 10.3390/ijms20061501 (PMC6471217; doi:10.3390/ijms20061501)
Supplement: Supplementary file 1 [file ijms-20-01501-s001.pdf]

Supplementary Material

Metabolic inhibition induces transient increase of L-type Ca^{2+} current in human and rat cardiac myocytes

Rimantas Treinys, Giedrius Kanaporis, Rodolphe Fischmeister and Jonas Jurevičius*

*** Correspondence: Jonas Jurevičius: jonas.jurevicius@lsmuni.lt**

Table 1

The effect of metabolic inhibitors on isoprenaline stimulated I_{CaL}

**Pharmacol. agent (µmol/L)**	**Increase of I_{CaL} in HAM**	**Suppression of I_{CaL} in HAM**	**Increase of I_{CaL} in HVM**	**Suppression of I_{CaL} in HVM**	**Increase of I_{CaL} in RVM**	**Suppression of I_{CaL} in RVM**
FCCP (0.1)	9.0±2.8% (n=3)	55.8±9.8% (n=4)	24.8±5.3% (n=3)	42.5±3.5% (n=6)	20.2±2.7% (n=12)	42.4±4.6% (n=12)
DNP (100)	–	–	5.0±0.9% (n=5)	44.8±4.4% (n=9)	–	–
Ant A (10)	–	–	–	–	8.3±2.3% (n=5)	35.5±1.9% (n=5)
Roten (30)	–	–	–	–	11.4±0.2% (n=3)	28.2±7.6% (n=3)

HAM – human atrial myocytes, HVM – human ventricular myocytes,
RVM – rat ventricular myocytes, Ant A – antimycin A, Roten – rotenone.

Supplementary Material

Metabolic inhibition induces transient increase of L-type Ca^{2+} current in human and rat cardiac myocytes

Rimantas Treinys, Giedrius Kanaporis, Rodolphe Fischmeister and Jonas Jurevičius*

*** Correspondence: Jonas Jurevičius: jonas.jurevicius@lsmuni.lt**

Table 2
Clinical characteristics of patients

**Patient data**	
Age range (years)	14 – 81
Mean age (years)	63
Female, *n*	9
Male, *n*	7
Total, *n*	16
**Surgical intervention**	
Aortic valve surgery[a], *n*	5/16
Mitral valve surgery[a], *n*	8/16
CABG surgery[a], *n*	9/16
Bentall operation, *n*	1/16
**Origin of specimen**	
Right atrial appendage, *n*	7/16
Left atrial appendage, *n*	1/16
Left ventricle apex, *n*	5/16
Interventricular septum, *n*	2/16
Left ventricle papillary muscle, *n*	1/16

[a] Some patients underwent both valve surgery and coronary bypass graft surgery. One patient underwent aortic valve, mitral valve and tricuspid valve surgery.

Supplementary Material

Metabolic inhibition induces transient increase of L-type Ca^{2+} current in human and rat cardiac myocytes

Rimantas Treinys, Giedrius Kanaporis, Rodolphe Fischmeister and Jonas Jurevičius*

*** Correspondence: Jonas Jurevičius: jonas.jurevicius@lsmuni.lt**

Transient increase of LTCCs current in isoprenaline stimulated rat cardiac myocyte during metabolic inhibition

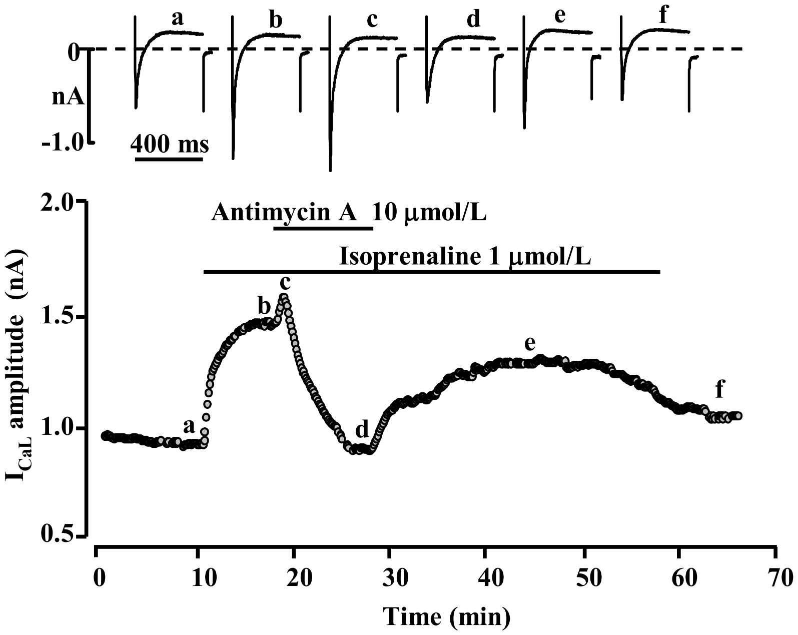

Supplementary Figure S1. Effect of antimycin A on ISO-stimulated I_{CaL} in rat ventricular cell. Traces of I_{CaL} shown on top were recorded at the times indicated by the corresponding letters on the main graph.

Supplementary Material

Metabolic inhibition induces transient increase of L-type Ca^{2+} current in human and rat cardiac myocytes

Rimantas Treinys, Giedrius Kanaporis, Rodolphe Fischmeister and Jonas Jurevičius*

*** Correspondence: Jonas Jurevičius: jonas.jurevicius@lsmuni.lt**

The time dependent inactivation of LTCCs current in isoprenaline stimulated rat cardiac myocyte during metabolic inhibition

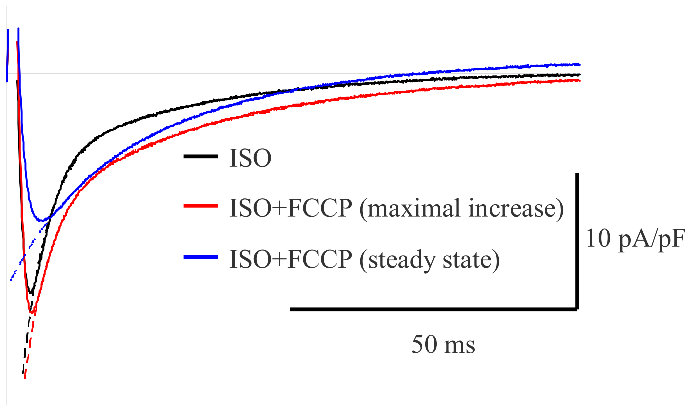

Supplementary Figure S2. Traces of I_{CaL} in isoprenaline (ISO, 1 µmol/L) stimulated (black line) rat cell during FCCP (0.1 µmol/L) induced maximal stimulation (red line) and suppression (blue line). Dashed lines represent double exponential fits of I_{CaL}s decay.

Supplementary Material

Metabolic inhibition induces transient increase of L-type Ca^{2+} current in human and rat cardiac myocytes

Rimantas Treinys, Giedrius Kanaporis, Rodolphe Fischmeister and Jonas Jurevičius*

*** Correspondence: Jonas Jurevičius: jonas.jurevicius@lsmuni.lt**

Suppression of cytosolic Ca^{2+} release

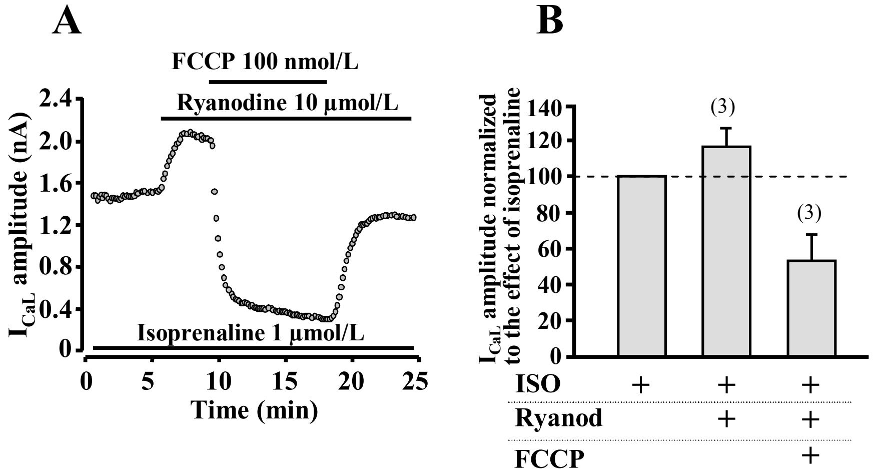

Supplementary Figure S3. Effect of FCCP on isoprenaline stimulated I_{CaL} in rat cardiomyocytes after suppression of cytosolic Ca^{2+} release. (A) A typical experiment representing the effect of FCCP on I_{CaL} in ISO-stimulated cell during exposure to ryanodine. (B) Peak amplitude of I_{CaL} during exposure of ISO-stimulated rat ventricular cells to FCCP in the presence of ryanodine. Values are presented as means ± SEM for the number of cells indicated in parentheses.

Supplementary Material

Metabolic inhibition induces transient increase of L-type Ca^{2+} current in human and rat cardiac myocytes

Rimantas Treinys, Giedrius Kanaporis, Rodolphe Fischmeister and Jonas Jurevičius*

*** Correspondence: Jonas Jurevičius: jonas.jurevicius@lsmuni.lt**

Facilitation of I_{CaL} in isoprenaline stimulated rat cardiac myocyte

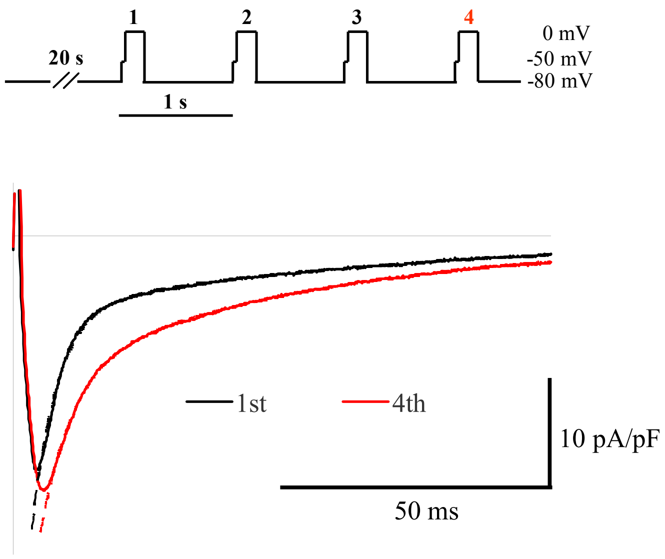

Supplementary Figure S4. Depolarization protocol for induction of I_{CaL} facilitation shown on top. Traces of I_{CaL} in isoprenaline (1 µmol/L) stimulated rat cell during application of 1 Hz stimulus. Superimposed are currents evoked at the 1st (black line) and 4th (red line) stimulations. Dashed lines represent double exponential fits of I_{CaL}s decay.

Supplementary Material

Metabolic inhibition induces transient increase of L-type Ca^{2+} current in human and rat cardiac myocytes

Rimantas Treinys, Giedrius Kanaporis, Rodolphe Fischmeister and Jonas Jurevičius*

*** Correspondence: Jonas Jurevičius: jonas.jurevicius@lsmuni.lt**

Stability of I_{CaL} over time in rat ventricular myocyte

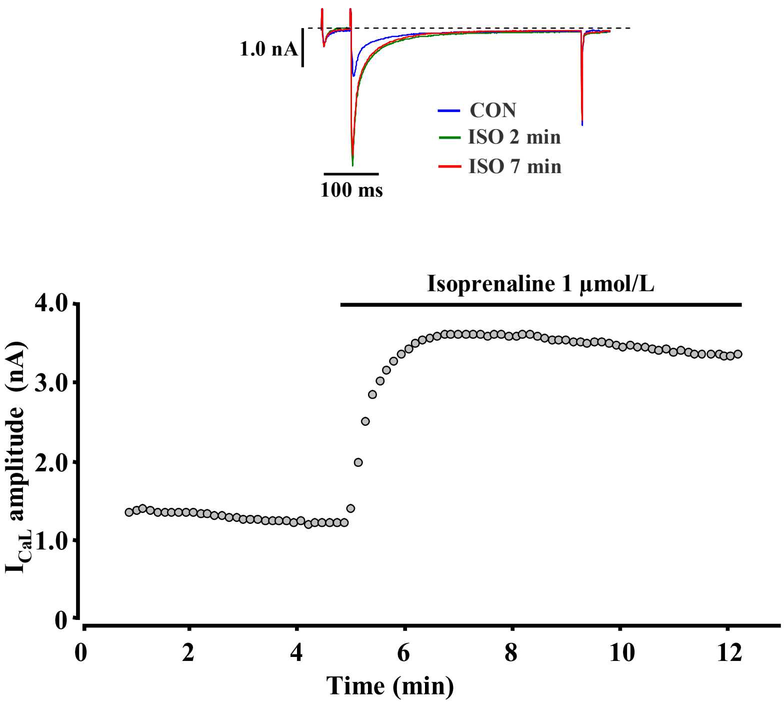

Supplementary Figure S5. Effect of isoprenaline on I_{CaL} in rat ventricular cell. Only run-down of the I_{CaL} was registered in control conditions and during β-adrenergic stimulation by isoprenaline (ISO), and no spontaneous increase in I_{CaL} was detected.
Traces of I_{CaL} shown on top were recorded in control conditions and during stimulation by ISO.
